# On the mechanism of piezoresistance in nanocrystalline graphite

**DOI:** 10.3762/bjnano.15.34

**Published:** 2024-04-08

**Authors:** Sandeep Kumar, Simone Dehm, Ralph Krupke

**Affiliations:** 1 Institute of Nanotechnology, Karlsruhe Institute of Technology, Kaiserstr. 12, 76131 Karlsruhe, Germany; 2 Institute of Quantum Materials and Technologies, Karlsruhe Institute of Technology, Kaiserstr. 12, 76131 Karlsruhe, Germany; 3 Institute of Materials Science, Technische Universität Darmstadt, 64287 Darmstadt, Germany

**Keywords:** grain boundary, nanocrystalline graphene, strain sensor, Raman, tunneling and destruction

## Abstract

Strain sensors are sensitive to mechanical deformations and enable the detection of strain also within integrated electronics. For flexible displays, the use of a seamlessly integrated strain sensor would be beneficial, and graphene is already in use as a transparent and flexible conductor. However, graphene intrinsically lacks a strong response, and only by engineering defects, such as grain boundaries, one can induce piezoresistivity. Nanocrystalline graphene (NCG), a derivative form of graphene, exhibits a high density of defects in the form of grain boundaries. It holds an advantage over graphene in easily achieving wafer-scale growth with controlled thickness. In this study, we explore the piezoresistivity in thin films of nanocrystalline graphite. Simultaneous measurements of sheet resistance and externally applied strain on NCG placed on polyethylene terephthalate (PET) substrates provide intriguing insights into the underlying mechanism. Raman measurements, in conjunction with strain applied to NCG grown on flexible glass, indicate that the strain is concentrated at the grain boundaries for smaller strain values. For larger strains, mechanisms such as grain rotation and the formation of nanocracks might contribute to the piezoresistive behavior in nanocrystalline graphene.

## Introduction

Flexible strain sensors are an important factor in moving from rigid to flexible electronics. Graphene, because of its interesting inherent properties, has found its way in many applications [1–3]. In particular, it is a promising alternative material as a transparent and conductive coating for future flexible electronics. This is because the relative change in resistance of graphene for similar values of applied strain (4%) is just 50%, which is two orders of magnitude lower than that of the flat-screen material indium titanium oxide (ITO). Indeed, from a theoretical point of view, change in resistance due to strain or piezoresistivity in graphene is expected to be small because the displacement of the Dirac point occurs in continuous *k* space, and strain-induced lattice distortions do not change the local band structure up to 20% strain [4]. In contrast, because of the quantized *k* space in carbon nanotubes, uniaxial strain can induce band opening or closing. Nevertheless, strain-induced resistance modulation in graphene is by far not zero, and reported values for relative resistance changes vary between 0.1% and 50% at 3% strain [5–8]. Although several works report the enhancement of piezoresistance in graphene, it is still unclear which factors influence this property. A theoretical work by Kumar et al. suggested that grain boundaries can affect piezoresistance in graphene [9]. This result seemed unexpected since Dirac particles should undergo Klein tunneling at barriers without adding up to the total resistance. However, the theoretical modeling shows that the modulation of the transport gap under strain is sensitive to the degree of asymmetry of the grain boundaries. While the symmetric grain boundaries remain metallic in the presence of uniaxial strain, the transport gap of the asymmetric semiconducting grain boundaries can be considerably increased in the presence of strain. Hence, the asymmetric metallic grain boundaries undergo a metal–semiconductor transition in the presence of strain. This effect could open a way to utilize grain boundaries in graphene for fabricating highly sensitive transparent strain sensors.

So far, the growth of specific grain boundaries in graphene has not been reported. Also, most research activities aim at the chemical vapor deposition (CVD) synthesis of monocrystalline graphene free of grain boundaries [10–12]. Methods to detect and visualize grain boundaries and dislocations are currently under development [13–14]. This leads to the situation that the role of grain boundaries for graphene-based sensing of strain, pressure, and motion has not been explored and remains unresolved [15–18], although in CVD graphene the domain size is typically of the order of a few micrometers [11]. We speculated that if grain boundaries are responsible for piezoresistivity in graphene, then the gauge factor should be enhanced if one reduces the grain size to a few nanometers.

Nanocrystalline graphene (NCG) is graphitic material with a crystal size of nanometers and, therefore, an excellent candidate for piezoresistance devices. Also, wafer-scale synthesis of NCG has already been achieved by Zhang et al. and modified by Riaz and co-workers [19–20]. Thickness-controlled growth of NCG was exploited to utilize it as an efficient broadband photodetector [21–22]. The preliminary work on the piezoresistivity of NCG looked promising but was limited regarding the applied strain because of rigid SiO_2_/Si substrates. In this work, we focus on the piezoresistance measurements in NCG at larger strain values. Initially, a two-point bending setup is described, which was constructed in-house and automated using Python. Then, sheet resistance measurements under externally applied strain are discussed. Raman spectroscopy of the NCG under strain is studied, which gives insights into the distribution of strain in the film. Utilizing electrical and optical properties, a mechanism for piezoresistance in NCG is proposed. The work included here is a part of the PhD thesis completed by the first author S. Kumar [23].

## Results and Discussion

**Figure 1:**
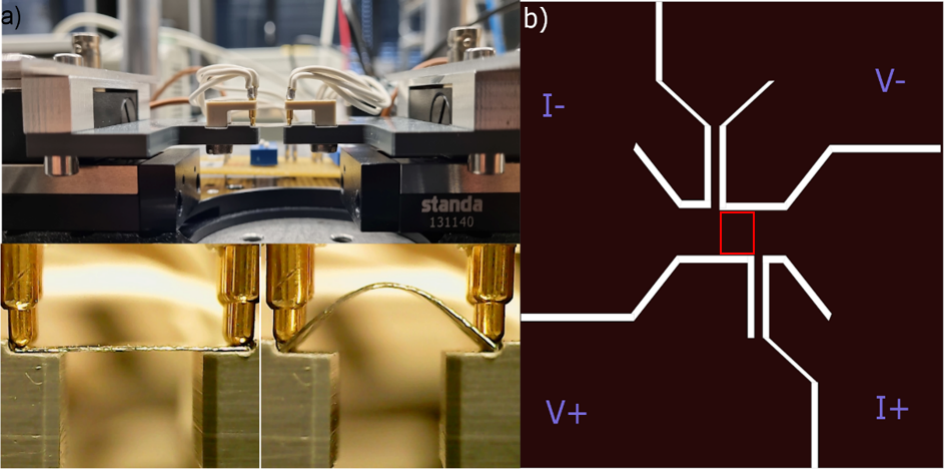
(a) Two-point bending fixture showing two instances of a substrate during measurement. (b) Patterned NCG structure for piezoresistance measurements. The red square marks the active device area. White lines are regions where NCG has been etched.

The two-point bending fixture, which was constructed to impart external strain and simultaneously perform sheet resistance measurements and Raman spectroscopy, is shown in Figure 1a. Two instances of zero and non-zero strain are also depicted. The setup has two stepper motors acquired from Standa Inc. The contacts on the NCG were made by gold-coated spring pressure contacts, which were then connected to a BNC connector and a Keithley 2636A device. The substrate holder and contacts holder were machined and attached to the stepper motor as shown in Figure 1a. A detailed description of the setup has been given by Kumar [23]. The complete setup was automated via self-programmed Python code. To completely eliminate any strain-induced changes in the contacts, the NCG was patterned such that the NCG itself is used as a contacting electrode (shown in Figure 1b). The area marked with a red square in Figure 1b (2 mm × 2 mm) is the active device area for sheet resistance measurements on a substrate of 10 mm × 10 mm area. Thin NCG constrictions at the end of the active device area were used to measure the potential drop across the device area. The measurements were done in constant current mode, and the voltage drop across the squared central area was measured at each strain value.

**Figure 2:**
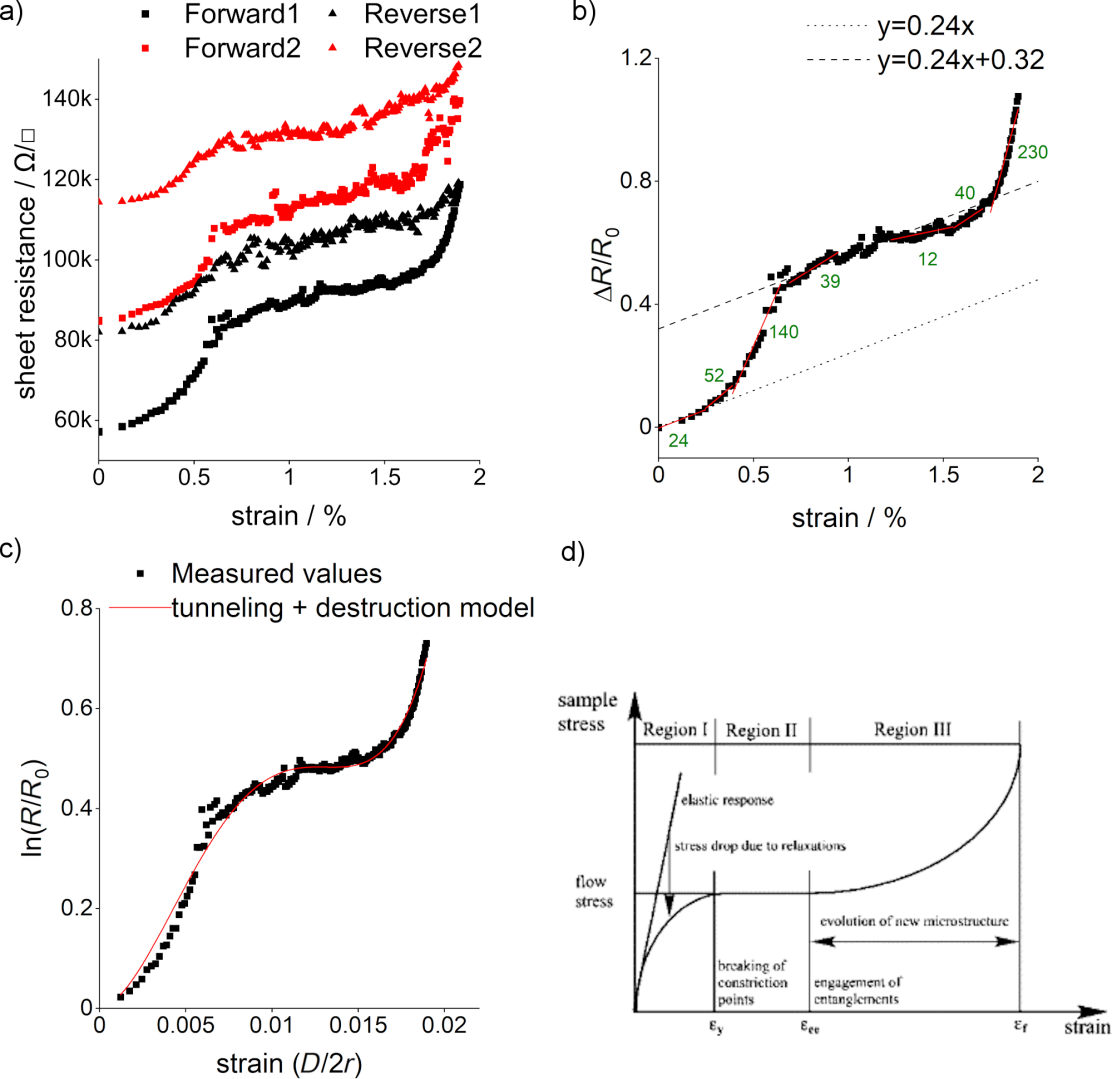
(a) Sheet resistance vs strain curve for NCG shown for two subsequently measured cycles of bending. (b) Δ*R*/*R*_0_ specifying GFs. The two parallel lines indicate similar GFs in two strain regimes. (c) Logarithm of normalized resistance vs strain curve for NCG with tunneling and destruction model [24]. (d) Typical stress–strain curve of amorphous polymer PMMA film (reprinted from [32], Polymer, Vol. 44, Issue 19, by Z. H. Stachurski, “Strength and deformation of rigid polymers: the stress–strain curve in amorphous PMMA“, pages 6067–6076, Copyright (2003), with permission from Elsevier. This content is not subject to CC BY 4.0.)

NCG was grown by spin coating S1805 at 4000 rpm; subsequently, it was transferred onto a 100 μm thick PET substrate. For more details please see the Experimental section. Measured piezoresistance curves (with forward and reverse sweep) for a 5 nm thick patterned NCG film on the PET substrate are shown in Figure 2a. Sheet resistance values are plotted against tensile strain up to 2%, which is an order of magnitude larger than in our previous work (max 0.1%) [19]. We could reproduce the previously observed gauge factor (GF) of ca. 24 at very low strain where the sheet resistance increases linearly with strain (<0.3%); however, in the extended-strain region we observe now a super-linear behavior, which evolves into a linear region from 0.7% to 1.6% strain, before entering a super-linear regime beyond 1.6% strain. The GF values from the the plot of normalized change in resistance vs strain (Figure 2b) also depict how the rate of resistance change with strain drops and then increases again. Interestingly, the GF is similar below 0.3% and in the region between 0.7% and 1.6% strain, indicating a similar origin of piezoresistance (shown by two parallel lines in Figure 2b). The overall shape of the curves is reproducible and shown here for two strain cycles. However, a hysteresis is observed between forward and reverse sweeps, indicating that structural changes in the films occur, which are in part irreversible.

**Figure 3:**
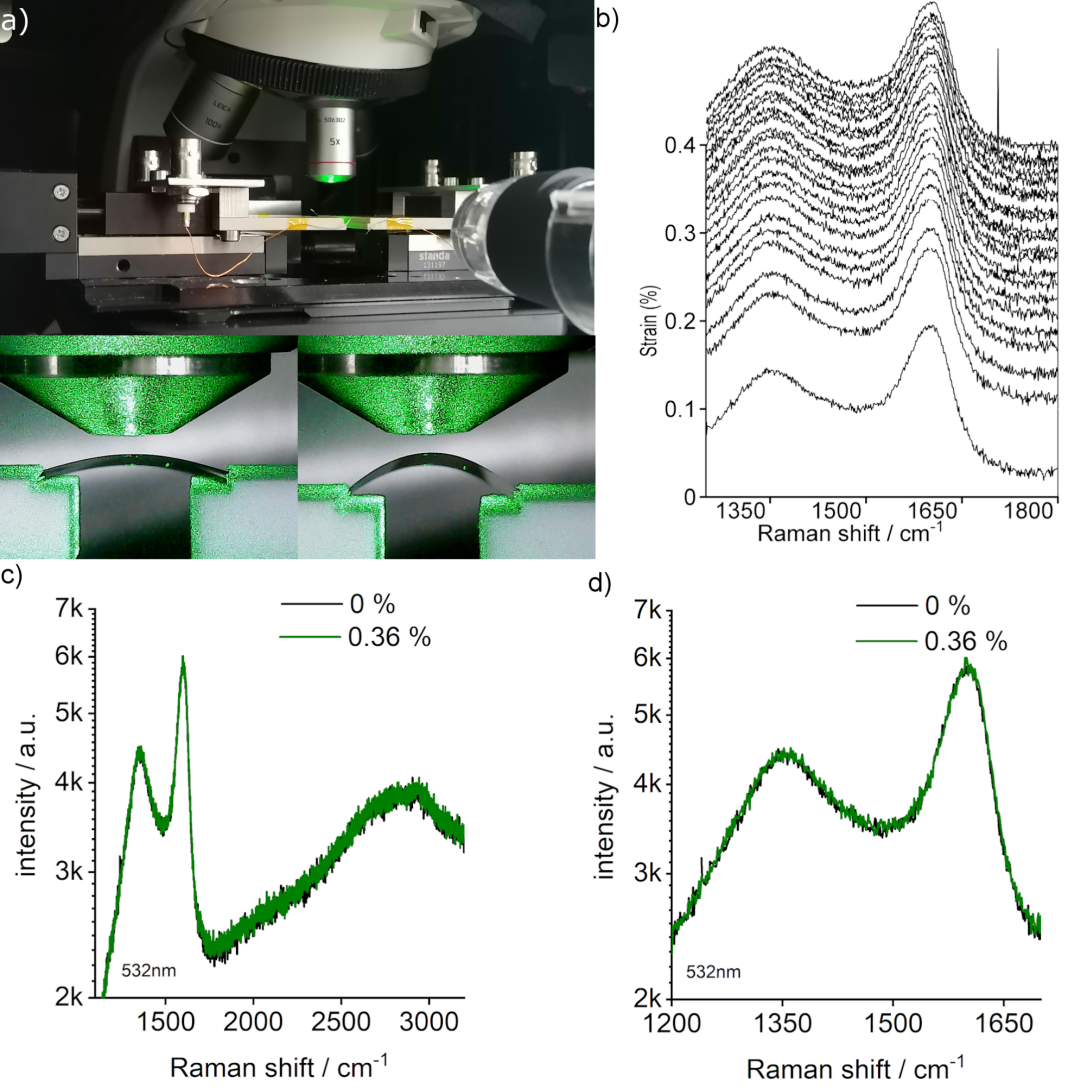
(a) Piezoresistance measurement setup enabling in situ Raman measurements under strain. (b) Raman spectra of NCG on glass with increasing strain from 0% (bottom) to 0.36% (top). Curves were shifted for clarity. (c) Comparison of full-range Raman spectra for 0% (black) and 0.36% (green) strain. (d) Comparison of Raman spectra focused on D and G peaks for 0% (black) and 0.36% (green) curves.

To gain insights into the strain distribution in the strained NCG, we performed in situ Raman measurements with strain as shown in Figure 3a. The flexible glass was preferred for the Raman measurements because the spectra of NCG cannot be resolved on PET as a result of a strong Raman signal of the substrate itself. The 50 μm thick flexible glass was acquired from Schott. The glass loses its flexibility at 600 °C and also in water [23]. To keep the flexibility, the NCG film was grown on both sides of the glass substrate. There are three reasons for that. First, the negative thermal expansion coefficient of NCG prevents the release of stress initially present in the glass [25]. Second, the film protects the glass from any corrosion from water if the transfer is required on glass in an aqueous medium [26]. Third, NCG fills the cracks present at the edges during spin coating the polymer and inhibits their propagation during the bending of the substrate.

Raman spectroscopy is a powerful method to detect strain in graphene, which can be determined from the analysis of the peak position of the 2D and G modes [27]. For the Raman measurements, the bending setup was installed in a Renishaw inVia Raman microscope, operated at 532 nm excitation wavelength (Figure 3a) with a 100× objective. Concado et al. [28] reported the general equation for calculating the crystallite size based on the full width at half maximum (FWHM) and intensity ratios of D and G peaks. Similar to our previous work [19], we use the FWHM of D and G peaks here at zero strain to calculate the crystallite size. The G peak gives a crystallite size of 2–3 nm, and the D peak corresponds to a crystallite size of 4–5 nm. TEM studies also done in our previous work on NCG gave an average domain size of 3 nm. We therefore report a grain size range of 2–5 nm for the NCG film synthesized for this study.

Figure 3b shows the Raman spectra as a waterfall plot for strain values up to 0.36%. Figure 3c,d shows a comparison between the spectra taken at 0% and 0.36% strain. Further measurements with increased strain could not be taken due to the failure of the glass substrate. Interestingly, no prominent changes in peak positions, widths, or intensity could be detected, inferring that the strain within the grains remains constant even though the externally applied strain increased to ca. 0.4%.

In an attempt to model piezoresistance in NCG, we have used the tunneling + destruction model for composite materials [24]:


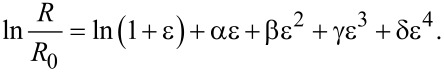


The model with five free parameters was fitted to the data as shown in Figure 2c, and the fit parameters are given in Table 1. The model was initially given for a matrix in which conducting particles are dispersed in a polymer matrix and are separated by tunnel junctions. In this model, the conductivity in the film is determined by the number of conductive paths, *N*, and the tunneling distance, *d*. The model has been used to explain the piezoresistance for several composite materials [29–30].

**Table 1:** Value of extracted parameters by fitting from Figure 2c.

Parameter	Value

α	5
β	1.55 × 10^4^ ± 1.8 × 10^2^
γ	−1.63 × 10^6^ ± 2.5 × 10^4^
δ	4.6 × 10^7^ ± 8.6 × 10^5^

Zhao et al. [24] used the model to explain the piezoresistance in nanographene films, although the material is comparable to ours and not a composite material in the original sense. NCG can be considered as a matrix of grains and grain boundaries (GBs), where the grains are separated by the GBs and have different resistivities [31]. Hence, the tunneling + destruction model might indeed be an appropriate physical representation of the nano/microstructure of NCG. The model explains that at lower strain values, only the tunneling distance *d* increases, but *N* remains constant. Whereas at larger strain values, *d* and *N* both change; therefore, the GF increases at those values. Our Raman measurements indicate that at least up to 0.4% strain, no strain is experienced by the grains, which would mean that all the strain energy provided externally ends up at GBs leading to movements of dislocations at GBs or fractures at GBs. Fitting the tunneling + destruction model to our data, we find that the initial tunneling distance has a value of 3.6 nm, which is comparable to the nanographene films fabricated by Zhao et al. (3.4 nm) [24]. The destruction of conduction channels as part of the tunneling + destruction model then takes place at higher strain values and eventually leads to partial irreversibility, which can be observed as an offset between the first and second trace in Figure 2a. Nevertheless, the overall shape of the second curve is similar to the first one. Figure 2d shows a typical stress vs strain curve for a polymer film [32]. The trend of the curve looks similar to the resistance vs strain curve for NCG in Figure 2a. Since polymers are insulators, literature on resistance vs strain for such films does not exist. However, it depicts how the stress drops because of strain relaxation in such films. Since resistance is directly proportional to strain in the tunneling + destruction model, one can think that the resistance of such films would also drop. Based on strain relaxations, therefore, one can correlate NCG and a polymer material and potentially give insight into how NCG behaves in the plateau region of the resistance vs strain curve.

It is important to note that many works that have reported piezoresistance in NCG have recorded data at comparably low strain values and have not observed the plateau-like region as reported here, where the gauge factor is similar to the gauge factor at very low strain [24,33]. A plateau-like region has neither been observed in nanocrystalline graphite [33], amorphous carbon films [34], nor in metallic films [35]. The mechanism that leads to an increase of resistance in amorphous carbon and gold films at large strain is crack formation. Also, in NCG, which is full of GBs and defects, crack formation and propagation have to be considered [36]. Assuming nanocrack formation at the GBs, we could understand the entire piezoresistance curve in the following way. The increase in resistance at lower strain (0.3%) would be determined by the piezoresistance of the GBs, as studied by Kumar and co-workers [9]. Beyond this strain value, the resistance of the film increases rapidly, which can be understood by nanocrack formation at certain GBs [37]. This phenomenon occurs up to 0.7% strain and then stops. The reason could be that the remaining GBs are stronger than the ones that fractured between 0.3% and 0.7% strain. The plateau after 0.7% strain indicates that although the strain is increased, no crack formation occurs. In addition, the slopes of the resistance vs strain curve at smaller strain and the plateau region are similar (Figure 2b), indicating a similar piezoresistance mechanism in both strain regions. This can be understood by strain relaxation occurring at GBs by crack formation and bond movement, as well as reformation and changes in the microstructure of the film [37]. After the plateau (strain > 1.6%), the resistance once again increases, and a new set of nanocracks start to form at different GBs. The second cycle of the resistance vs strain measurement (Figure 2a, red curve) starts from a resistance value that is equivalent to the resistance at 0.7% strain in the first cycle. This indicates that the nanocracks formed in the NCG film between 0.3% to 0.7% strain are non-reversible cracks. However, the cracks formed between 1.6% and 2.0% strain are partially reversible, and the resistance values are almost recovered. This is possible through bond reformations by formation of pentagon–heptagon pairs due to their low formation energy [38–39]. In addition, the formation of nanocracks appears not so pronounced in the second cycle, as can be seen from the change in resistance between 0.3% and 0.7% and between 1.6% and 2.0% strain, and is likely due to cracks already formed in the first cycle.

**Figure 4:**
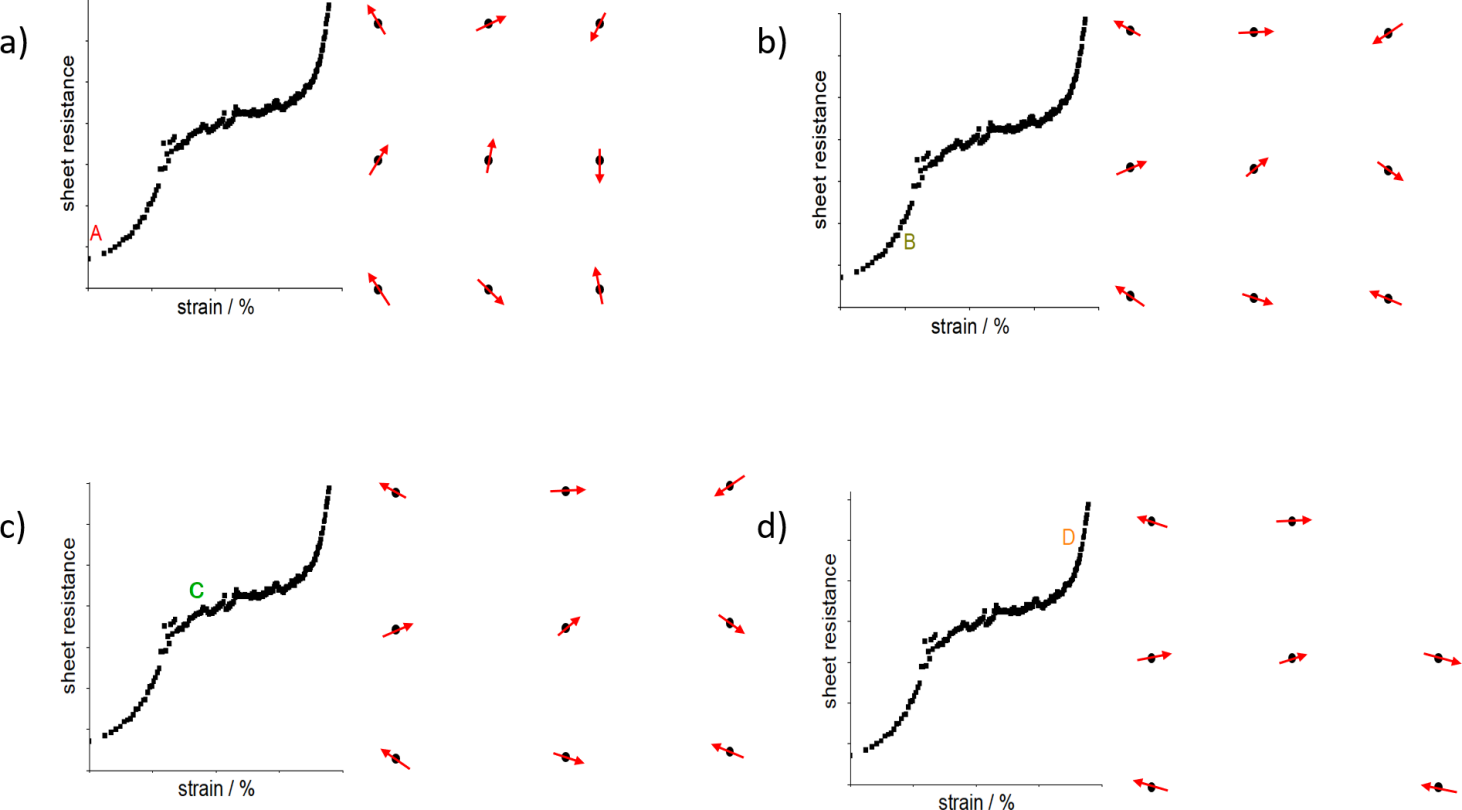
Correlation between transport in NCG films and grain rotation and reorientation under strain. Black dots represent the grain position, and arrows represent the long axis of non-spherical grains (a) at zero strain (marked as point A in the curve), (b) at point B in the curve, (c) in the plateau region marked as point C, and (d) at point D in the curve showing missing grains as rupture in the film.

Another process that might be considered for strain relaxation is grain rotation as shown in Figure 4. The rotation of grains during the straining of metal films has been studied extensively [40–41]. The effect is prominent for metal films with smaller grain sizes and diminishes with larger grains. The mechanism has been proposed as a cause of plastic deformation in metals. Wang et al. showed for a platinum nanocrystalline film [42] that the rotation due to strain at room temperature does not occur because of cross-grain gliding, GB sliding, or diffusional creep processes. The rotation occurs because of a change in the content of GB dislocations, which can change the GB angle between the grains (Frank–Bilby equation).

We cannot completely exclude that GB rotation occurs to some extent also in strained nanocrystalline graphene. Figure 4 shows a schematic of a NCG film under strain correlated with the transport of the film. The black dots represent the grain position, and the arrows represent the long axis of non-spherical grains. At the beginning, marked as point A on the curve, the grains are randomly oriented. As the strain is applied, the grains start to move apart, which is visible as an increase in resistance values between 0% and 0.3% strain [24], also confirmed by Raman measurements under strain (Figure 3b–d). At 0.3% to 0.7% strain, grain rotation and irreversible changes in the microstructure occur. This is seen by a sharper increase in resistance and a larger GF value, which corresponds to point B in Figure 4b showing grains moving apart in combination with grain rotation. At the plateau region (0.7%–1.6%), as explained before, the slope of the resistance vs strain curve is equivalent to the lower strain region (0%–0.3%) indicating a similar piezoresistance mechanism of grains moving apart and increasing tunneling distance. This is shown as point C in the transport in Figure 4c, where the grains are locked and cannot rotate, and the increase in resistance only occurs because of increased distance between grains. Above 1.6% strain, a sharper increase in resistance indicates again grain rotation and reorientation, and fracture in the film shown by missing black dots and arrows in Figure 4d, corresponding to point D in the transport curve. The processes of grain movements (increase in distance between grains and rotation) would repeat if the strain values are increased further until the fracture of the film. The process of bond rotation and reformation is known in NCG films for the relaxation of stress at GBs [37]. When the application of strain is reversed, irreversible changes occurring in the film by grain rotation results in a permanent increase in initial resistance seen by a hysteresis (blach and red curves in Figure 2a) and an offset in the second cycle (red curve in Figure 2a) [43]. Although the processes are different, there is a competition between bond breaking and rotation at certain strain values; the kinetically favorable process occurs in alternating sequences related to thermal and stress fluctuations inducing nanocrack formation [37,44]. Yang et al. [45] have shown a simulation of the stress vs strain behavior in NCG films at different temperatures and strain rates. Interestingly, the curve looks similar to the resistance vs strain curve in this work. A deviation from the linear behavior into a plateau is observed at larger strain, owing to plastic deformations of the NCG film. However, a sharp increase in stress after the plateau region is not observed. This can be understood by stress relaxation due to fractures in the film at higher strain, which is visible in resistance vs strain curves as a sharp increase in the resistance. As a last comment, Zhao et al. [24] reported that as they reduced the grain size from 25 to 8 nm, the GF increased from 11 to 600. Also, Simionescu et al. [33] reported a varying GF (50–250) for a strain range of 0%–1%. In this work, NCG with lower grain sizes has been obtained; however, the GF does not appear to further increase and remains comparable to the values of previously reported works. A comparison is tabulated in Table 2.

**Table 2:** Comparison of grain sizes of NCG with the corresponding GFs.

Grain size (nm)	Gauge factor (GF)	Strain range (%)	Reference

25	11	0 to 1	[24]
8	600	0 to 1	[24]
9	50–250	0 to 1	[33]
2–5	23	0 to 0.1	[19]
2–5	24–140	0 to 2	this work

## Conclusion

This study endeavors to further the understanding of the piezoresistance mechanism in NCG, employing a two-point bending setup to apply controlled strain. The strained NCG was analyzed electrically and optically, revealing three regimes in the sheet resistance vs strain curve. Examination of the results from optical and electrical measurements suggests that in the lower strain regime, the grains experience negligible effects, while the majority of strain is concentrated at the grain boundaries. Consequently, non-reversible cracks form at GBs. The second regime exhibits a superlinear dependence of sheet resistance on strain, indicating potential grain rotation and bond reformation, leading to a modified nano/microstructure. In the larger-strain regime, an exponential increase in sheet resistance vs strain signifies further partially reversible crack formation. To enhance understanding, a tunneling + destruction model was fitted, and parameters were extracted. While the paper offers an overview of piezoresistance in NCG, a more in-depth study is imperative for a complete comprehension of the system’s complexity. In situ FTIR measurements could provide additional insights into changes in doping and defects with strain.

## Experimental

### Piezoresistance measurements

NCG was synthesized on a 300 nm SiO_2_/Si substrate by spin coating S1805 (1:10 dilution with propylene glycol methyl ether acetate, PGMEA) at 4000 rpm. The spin-coated Si/SiO_2_ substrate was loaded in a vacuum furnace and annealed at 600 °C for 10 h at 10^−6^ mbar. The measured thickness of the grown film was ca. 5 nm. The NCG film was then transferred onto a 100 μm thick PET substrate. For the transfer process, first, the NCG film on SiO_2_/Si was coated with 200 nm thick PMMA and put into 5 M NaOH solution at 80 °C. The NCG/PMMA film floats on the surface after the etching of SiO_2_. Using a clean glass wafer, the NCG/PMMA film was transferred from the NaOH solution to a clean water beaker and allowed to float on the top. The cleaning was repeated three times to ensure the no residues of NaOH remained on the NCG film. The film was then removed from the water using a PET substrate. After that, the substrate was left in air for drying. Next, a drop of PMMA was dripped on top of the film and allowed to spread and dry. This has been shown to be helpful in removing wrinkles formed during the transfer process [46]. The NCG film on the PET substrate was then patterned in the structure shown in Figure 1b using e-beam lithography. There were no metal films deposited on NCG, and the electrical contact was made between gold spring contacts and NCG directly. For Raman measurements, S1805 (1:10 dilution with PGMEA) was spin-coated on both sides of the flexible glass substrate at 4000 rpm to grow NCG on both sides of the glass. The substrate was then loaded into the vacuum furnace and treated similarly.

### Raman measurements

Raman measurements were done using a 100× objective at 0.6 mW laser power for 60 s integration time for each measurement. The same area on the NCG film was focused as to monitor and compare any changes occurring during straining the film.
